# Marked conduction time prolongation observed during atrial tachycardia originating from a giant left atrial appendage

**DOI:** 10.1002/joa3.13152

**Published:** 2024-10-23

**Authors:** Kazuhisa Matsumoto, Daisuke Kawano, Hitoshi Mori, Yoshifumi Ikeda, Ritsushi Kato

**Affiliations:** ^1^ Department of Cardiology Saitama Medical University, International Medical Center Hidaka Japan

**Keywords:** atrial tachycardia, catheter ablation, conduction delay, giant left atrial appendage

## Abstract

We experienced a rare case of atrial flutter originating from the giant left appendage (LAA). The local potential of the ablation catheter presented with a rare finding, appearing up to 185 ms earlier than the surface P‐wave in the distal LAA. With thoracoscopic LAA clipping, tachycardia was successfully controlled.

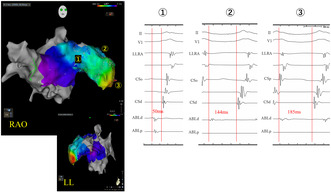

A 52‐year‐old woman was referred to our hospital for catheter ablation of atrial tachycardia (AT). The P‐wave on the 12‐lead electrocardiography (ECG) during tachycardia was negative in leads I/aVL, positive in V1, and bimodal in lead II (Figure 1). The tachycardia (cycle length 240–260 ms) occurred spontaneously once the electrode catheter was positioned and could not be reproducibly induced or terminated with serial atrial pacing. Serial atrial pacing from the low lateral right atrium (LLRA) and CSd during tachycardia revealed longer postpacing interval (PPI) than tachycardia cycle length. The P‐wave morphology on the 12‐lead ECG and the electrogram (EGM) sequence suggested a left atrial origin. Therefore, a transseptal approach was conducted for left atrial catheterization, and 3D activation map (CARTO®3, Johnson and Johnson, New Jersey) was performed using Octaray™ (Biosense Webster, CA, USA) catheter. The activation map showed focal activation pattern, identified the earliest activation site at the base of the LAA (Figure S1). Although detailed pacing study was not performed and the possibility of macro‐reentry within the LAA cannot be completely ruled out, the activation map showed a centrifugal pattern, suggesting a high likelihood of focal AT originating from the LAA. Therefore, we decided to perform ablation targeting the earliest activation site. Ablation at the site 50 ms earlier than the earliest activation site temporarily terminated the tachycardia, but it recurred immediately. Therefore, we mapped the further distal site of the LAA and found there was earlier local atrial potential. LAA angiography revealed that LAA was markedly enlarged with a septum‐like structure in its midsection (Figure 1B), where the catheter was trapped, making its manipulation difficult. We subsequently used Octaray™ catheter and ablation catheter for additional mapping. As we were unable to freely maneuver the catheter in the distal part of the LAA, we could only obtain 3D mapping images within the range where it could be operated. As the ablation catheter advanced from proximal to the distal portion of LAA, the local potential of the ablation catheter gradually preceded the surface P‐wave, up to 185 ms in the distal LAA (Figure 2, Figure S2, Video S1). Ablation at the earliest site using QDOT MICRO™ (Biosense Webster, CA, USA) catheter was unsuccessful due to wattage feedback from rapid temperature rise and a non‐QS pattern in the unipolar potential, indicating the absence of the true earliest site (Figure S3). The session was terminated without controlling the tachycardia. Subsequently, thoracoscopic LAA clipping (AtriClip™, AtriCure, OH, USA) was performed (Figure 3), and no tachycardia was observed thereafter.

**FIGURE 1:**
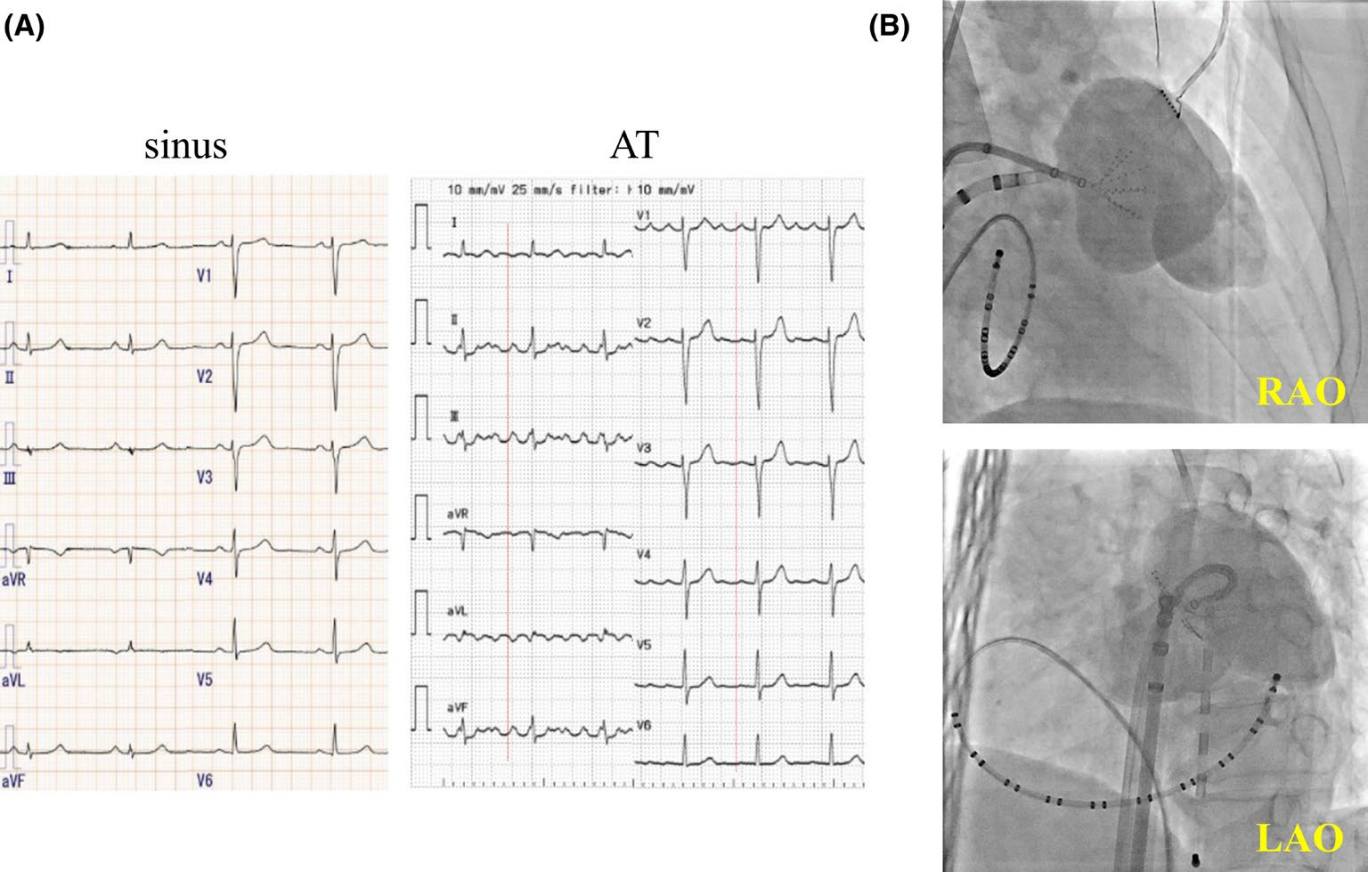
(A) Shows the electrocardiogram (ECG) during sinus rhythm (left) and tachycardia (right). During tachycardia (right), the P‐wave morphology is positive in lead V1, negative in leads I/aVL, and bimodal in leads II/III/aVF. The red line shows the beginning of P‐wave. (B) Illustrates the left atrial appendage angiography.

**FIGURE 2:**
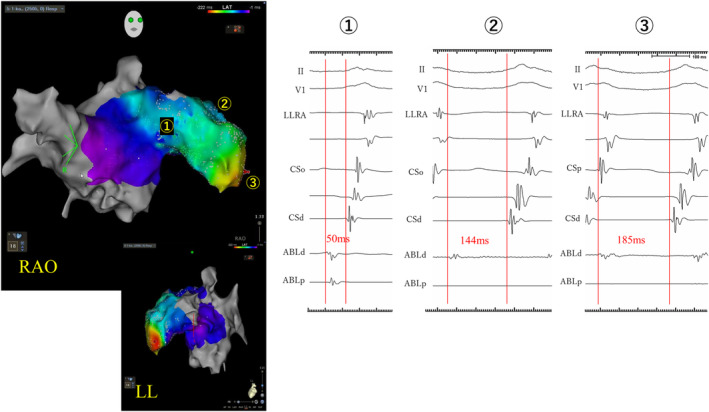
Shows the changes in the precedence of local potentials of the ablation catheter at each location within the left atrial appendage (LAA). Point ① shows a local potential preceding P‐wave of the surface by 50 ms. As the catheter advances distally, the local potentials precede by 144 ms (Point ②) and 185 ms (Point ③), respectively.

**FIGURE 3:**
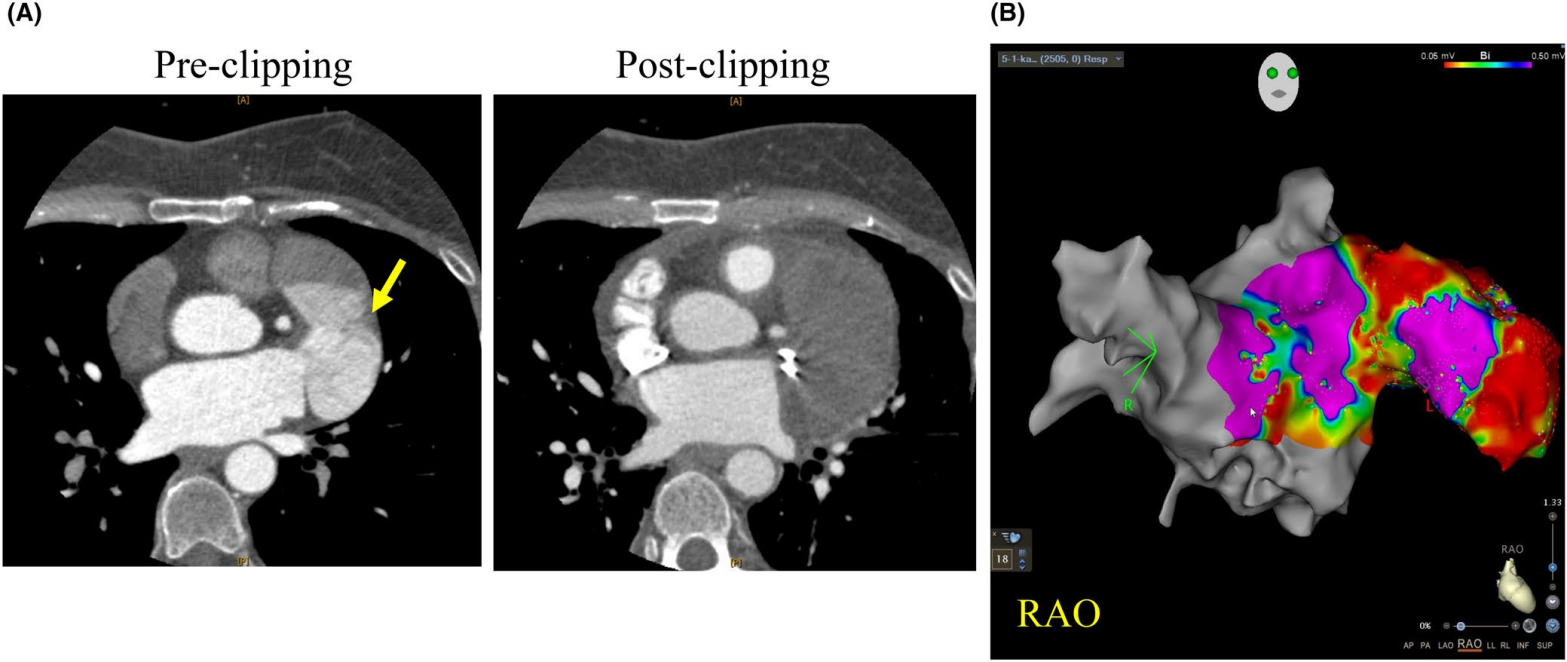
(A) Shows CT images before and after clipping. The midsection of the left atrial appendage (LAA) displays a septum‐like structure (left, yellow arrow). Postclipping, no contrast agent is observed entering the LAA (right). (B) Illustrates the voltage map. Low voltage areas are widespread at the base and distal parts of the LAA, except for the anterior portion of the midsection.

Atrial tachycardia (AT) originating from the LAA account for 2%–19% of cases. However, AT associated with a giant LAA, as observed in this case, is rare.^1^ Conduction from the distal LAA to the atrium, reflected as the P‐wave on the surface ECG, took 185 ms, yet the unipolar potential of the ablation catheter did not exhibit a typical QS pattern, suggesting the presence of even earlier potentials. Several studies have reported histological findings of fibrosis and wall thinning in LAA aneurysm.^2^, ^3^ Although histological evaluation was not performed in this case, the voltage map revealed that low voltage areas were widespread at the base and distal parts of the left atrial appendage, except for the anterior portion of the midsection (Figure 3B), suggesting that fibrosis had progressed, resulting in significant conduction delay of approximately 200 ms before being reflected in the P‐wave on the 12‐lead ECG. In this case, the P‐wave during sinus rhythm had a normal morphology, and the conduction delay in the LAA was not reflected. The conduction delay in the LAA became apparent only when tachycardia occurred. We considered that the extensive scar within the LAA, resulting in overall low voltage, may have prevented the conduction delay from affecting the P‐wave during sinus rhythm. Ablation success rates for tachycardias originating from the distal LAA are reported to be low (approximately 10%).^4^ In cases with a markedly enlarged LAA, catheter maneuverability in the LAA significantly decreases. Furthermore, since the distal LAA has blind‐end structure, it is prone to temperature rises leading to insufficient ablation because of temperature control. If endocardial ablation is unsuccessful, an epicardial approach or surgical intervention (resection or clipping) is recommended to mitigate risks such as perforation or phrenic nerve palsy. In this case, thoracoscopic clipping was performed from a minimally invasive perspective, and electrical isolation was achieved.^5^

We present a rare case of AT originating from a giant LAA with significantly prolonged conduction time within the LAA. Catheter ablation was ineffective, and the tachycardia was controlled with surgical LAA clipping.

## FUNDING INFORMATION

This report did not receive any specific grant from funding agencies in the public.

## CONFLICT OF INTEREST STATEMENT

All authors have no conflict of interest to disclose.

## ETHICAL APPROVAL

N/A.

## PATIENT CONSENT STATEMENT

Informed consent for the publication of this case report was obtained from the patient.

## Supporting information


**Figure S1.** Shows the initial activation map and the ablation site (earliest activation site, Red pin). This map does not cover the entire left atrial appendage.
**Figure S2.** Shows isochronal map during atrial tachycardia.
**Figure S3.** Shows the ablation site. At the distal LAA, after starting the ablation, the wattage immediately decreased due to temperature control, making it difficult to achieve sufficient ablation.

**Video S1.** Shows the propagation map within the LAA. The activation is propagating centrifugally from the distal of LAA.

## Data Availability

Data sharing is not applicable to this article as no data sets were generated or analyzed during the current study.

## References

[1] Combes S , Albenque JP , Combes N , Boveda S , Cardin C , Ciobotaru V , et al. An original management of focal atrial tachycardia originating from a giant left atrial appendage. HeartRhythm Case Rep. 2018;4(4):135–137. 10.1016/j.hrcr.2017.10.016 29755939 PMC5944058

[2] Nagai T , Higaki J , Okayama H . Cardiovascular flashlight. Atrial tachycardia in congenital left atrial appendage aneurysm: three‐dimensional computed tomography imaging with electro‐anatomical mapping. Eur Heart J. 2010;31(13):1590. 10.1093/eurheartj/ehq046 20179164

[3] Yan W , Xie Y , Cai P , Ma X . A giant left atrial appendage aneurysm with recurrent chest tightness and atrial tachycardia: multimodal imaging findings. Radiol Case Rep. 2023;18(3):805–808. 10.1016/j.radcr.2022.11.062 36589491 PMC9794895

[4] Yang Q , Ma J , Zhang S , Hu JQ , Liao ZL . Focal atrial tachycardia originating from the distal portion of the left atrial appendage: characteristics and long‐term outcomes of radiofrequency ablation. Europace. 2012;14(2):254–260. 10.1093/europace/eur302 21933799

[5] Benussi S , Mazzone P , Maccabelli G , Vergara P , Grimaldi A , Pozzoli A , et al. Thoracoscopic appendage exclusion with an atriclip device as a solo treatment for focal atrial tachycardia. Circulation. 2011;123(14):1575–1578. 10.1161/CIRCULATIONAHA.110.005652 21482976

