# Supplementary figures and images for: Marked conduction time prolongation observed during atrial tachycardia originating from a giant left atrial appendage
Source: J Arrhythm. 2024 Oct 23;40(6):1494–6. doi: 10.1002/joa3.13152 (PMC11632257; doi:10.1002/joa3.13152)

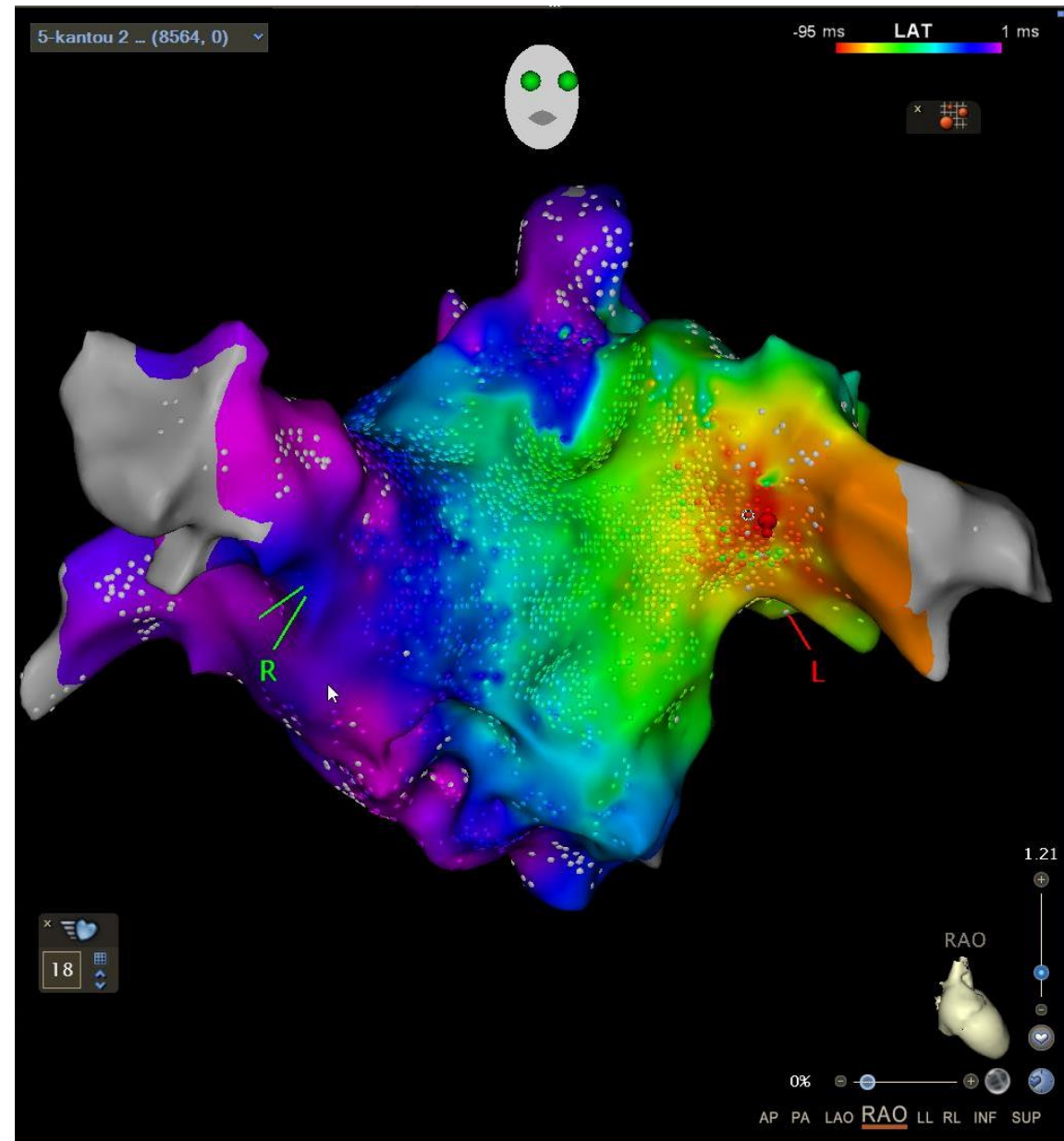

Supplemental Figure 1

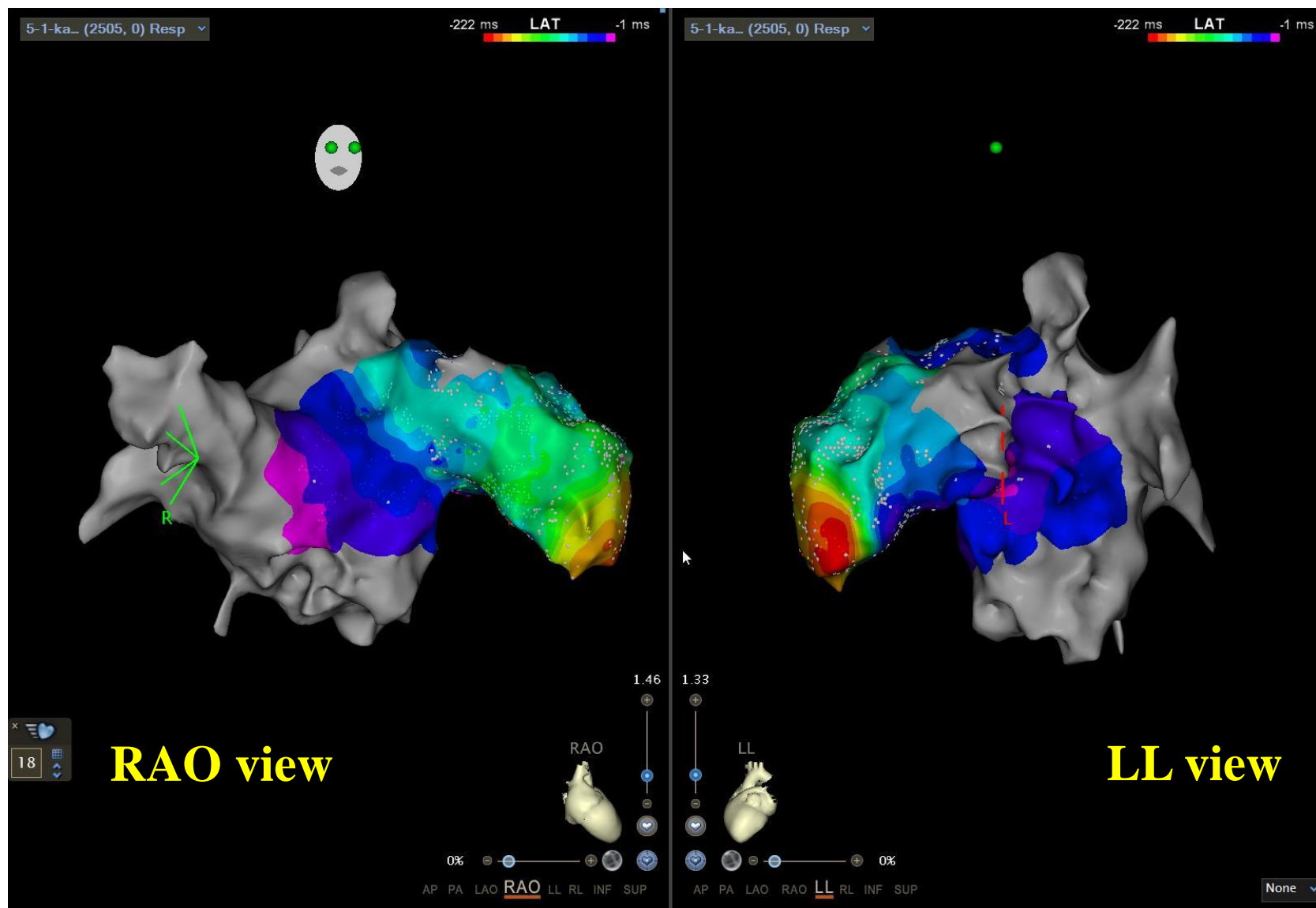

Supplemental Figure 2

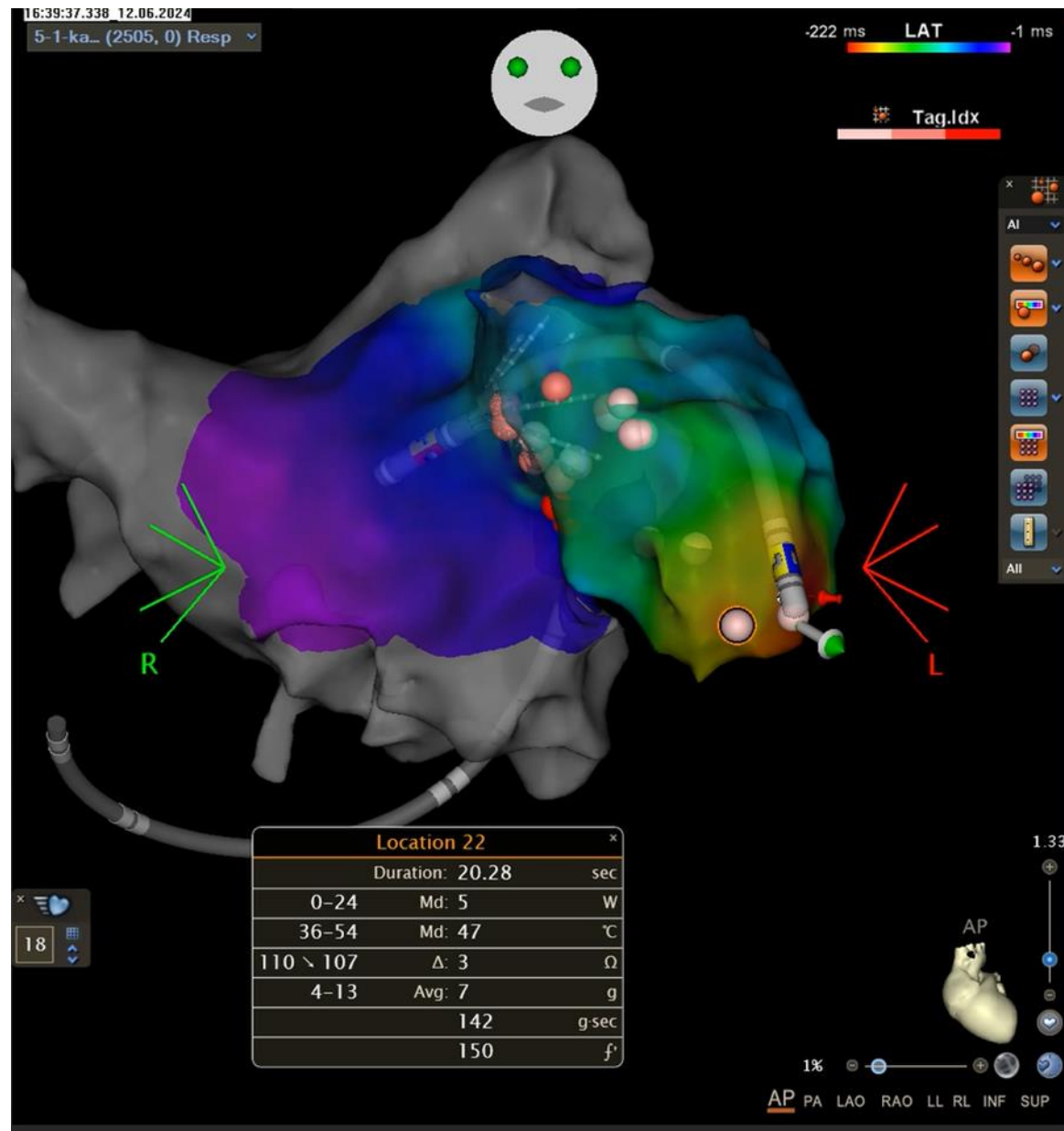

Supplemental Figure 3

Supplement: Supplementary file 1 — Figure S1. Shows the initial activation map and the ablation site (earliest activation site, Red pin). This map does not cover the entire left atrial appendage. Figure S2. Shows isochronal map during atrial tachycardia. Figure S3. Shows the ablation site. At the distal LAA, after starting the ablation, the wattage immediately decreased due to temperature control, making it difficult to achieve sufficient ablation. [file JOA3-40-1494-s001.pdf]
